# Editorial

**Published:** 1871-05

**Authors:** 


### Editorial.

The next number of The Examiner will contain a full account of the doings of the annual meetings of the American Association of Medical Editors, and of the American Medical Association, to be held in SanFrancisco during the first week in May, from the pen of the senior editor.

Wisconsin State Medical Society.—The next annual meeting of this Society will be held in Milwaukee, commencing on Wednesday, June 21, 1871, at 7 o'clock P.M.

Poisoning from Belladonna—Recovery.—Early on the morning of April 15th, I was called to see Hattie F.; 12 years of age; daughter of one of the prominent residents of the suburb of Oakland. She was suffering from a severe inflammation of the throat and pharynx, of a diphtheritic nature, accompanied with considerable swelling of the tonsils and glands of the neck. The following solution was ordered:

℞. Chlorate Potass.,———————ʒj.

Hydrochl. Acid,———————gtts. xij.

Tinct. Belladonna,———————ʒij.

Simple Syrup,———————℥j.

Water,———————℥iij. M.

A teaspoonful to be given every two hours.

I was sent for in great haste to see the patient again, in the afternoon of the same day. The message was not received, however, in time to reach there before 5½ P.M. She was then found to be in a very alarming state, with a high fever; pulse 140; face flushed; pupils widely dilated; mouth and fauces excessively dry; the mind was wandering, delirious; and there was constant nervous twitchings and startings.

It was at once apparent that she was under the influence of a large over-dose of belladonna, and the dark turbid appearance of the medicine, which if properly prepared should have been clear, and of a light amber color, confirmed the opinion that there had been a mistake in filling of the prescription. The parents had become very much alarmed at the condition of the child, but supposed that it was owing to an increase in the severity of the disease, and had continued to give the medicine regularly, the last dose having been given only a few minutes previous to my arrival. I at once ordered a solution consisting of the following:

℞. Chlorate Potass.,------------------------ʒj.

Sulph. Morph.,------------------------gr. j.

Simple Syrup,--------------------------℥j.

Water,-------------------------------℥iij. M.

A teaspoonful to be given every half-hour until three doses had been taken, and then continued every hour until the symptoms were relieved.

On the following day the patient was found to be considerably relieved, although the pupils were still somewhat dilated, and the mind a little wandering.

I took the vial containing the solution to the druggist's where it had been prepared, on the corner of Waupanseh Ave. and Cottage Grove Ave., and requested to see the prescription from which it had been put up. The prescription was plainly and distinctly written, exactly as given above. The clerk who filled the prescription was not present at the time, but it was quite evident, as well from the appearance of the mixture, as from its effects, that the *solid extract* of belladonna had been substituted for the tincture, as called for in the prescription. This would give nearly four grains of the belladonna to each dose of the mixture.

Five doses were given in all, making 19 grains that were administered to the child within 10 hours.

No permanent bad effects seem likely to result from the accident. On yesterday, the 19th, the patient had enjoyed considerable quiet sleep, was perfectly rational, and appeared to be rapidly progressing toward recovery.

### ILLINOIS STATE MEDICAL SOCIETY.

OFFICE OF THE PERMANENT SECRETARY,
*296 W. Monroe St., Chicago, Ill., April 19, 1871.*

The Twenty-first Annual Session of this Society will be held in the city of Peoria, on the third Tuesday in May, 1871, at 10 o'clock A.M.

The following committees are expected to report:

*On Practical Medicine*—Dr. H. A. Johnson, of Chicago, Chairman.

*On Surgery*—Dr. E. Andrews, of Chicago, Chairman.

*On Obstetrics*—Dr. DeLaskie Miller, of Chicago, Chairman.

*On Drugs and Medicines*—Dr. E. Ingalls, of Chicago, Chairman.

*On Criminal Abortions*—Dr. W. H. Byford, of Chicago, Chairman.

*On Ophthalmology*—Dr. H. H. Roman, of Springfield, Chairman.

*On Phthisis Pulmonalis*—Dr. S. W. Noble, of Bloomington, Chairman.

*On Otology*—Dr. S. J. Jones, of Chicago, Chairman.

*On Criminal Insanity*—Dr. A. McFarland, of Jacksonville, Chairman,

*On Idiocy*—Dr. C. T. Wilbur, of Jacksonville, Chairman.

*On Medical Uses of Carbolic Acid*—Dr. N. S. Davis, of Chicago, Chairman.

*On Cholera Infantum*—Dr. D. W. Young, of Aurora.

*On Membranous Croup*—Dr. D. Prince, of Jacksonville.

*On Gleet*—Dr. R. S. Higgins, of Vandalia.

*On Results in Fractures*—Dr. G. W. Phillips, of Dixon.

*On Intestinal Affections of Infants*—Dr. T. D. Fitch, of Chicago.

*On Diabetes*—Dr. W. D. Sterling, of Ionia.

*On Perineal Section*—Dr. E. Powell, of Chicago.

*On Duties of the Profession to the State Medical Society*—Dr. J. S. Whitmire, of Matamora.

*On Causes of Mortality after Amputation*—Dr. R. N. Isham, of Chicago.

Medical societies are entitled to one delegate for every five of their regular resident members, and one for every additional fraction of more than half this number.

The faculty of every regular medical college is entitled to two delegates.

Every chartered or municipal hospital is entitled to one delegate.

Any respectable physician, unable to attend as a delegate or permanent member, may attend as a *member by invitation*, on the recommendation of the Committee of Arrangements.

*Railroad Arrangements*—Arrangements have been made with all the railroads leading to Peoria, to return delegates and permanent members, on the certificate of the Secretary, for one-fifth fare, except the Chicago, Rock Island & Pacific Railroad. With this Company we have the following arrangement: Any member or delegate expecting to travel over this road to the Society, can do so on excursion or round-trip tickets, for sixty per cent. of the regular fare, by obtaining from me an order before leaving home, which, on being presented to the ticket agent at any station on the road, will entitle the holder to a round-trip ticket at the above rate.

I shall send to every member of the Society, that will probably go over this road, an order, which, if they do not wish to use, they may hand to any delegate or return to me.

T. D. FITCH.

---

MONEY RECEIPTS FROM MARCH 25, TO APRIL 25.—Dr. W. H. Massey, $3.00; Dr. J. H. Hudson, 2.00; Dr. J. S. Sweeny, 12.00; Dr. A. J. Miller, 9.00; Dr. S. McBride, 12.00; Dr. U. P. Stair, 12.00; Dr. Aug. Rhodes, 3.00; Dr. A. F. Chandler, 3.00; Dr. J. A. Adrion, 3.00; Dr. E. B. Woolcott, 6.00; Dr. S. A. McWilliams, 5.00; Dr. E. P. Cook, 6.00; Dr. John Higgins, 5.00; Dr. C. McAllister, 3.00; Dr. R. C. Moore, 6.00.

---

TREATMENT OF STRYCHNIA POISONING BY BROMIDE OF POTASSIUM.—Two cases have recently been reported, in which the bromide appears to have been successful as an antidote to strychnia—one in the *American Medical Journal*, the other in the *New York Medical Journal*. In the latter case, 90 grains were given every half-hour till the muscles were relaxed.

### Mortality for the Month of March, 1871.

| | | | | | |
|---|---|---|---|---|---|
| Accidents, drowned | 1 | Cyanosis | 2 | Lungs, congestion of | 8 |
| " burned in building | 1 | Cynanche trochealis | 2 | " hemorrhage of | 1 |
| " brain concussion | 1 | Debility, general | 4 | Malformation, general | 1 |
| " crushed by bridge | 1 | Diarrhœa and abscess | 1 | Manslaughter | 1 |
| " fall | 4 | " chronic | 2 | Measles | 11 |
| " " from buggy | 1 | Diphtheria | 8 | " and complications | 7 |
| " railroad | 2 | Diaphragnitis | 1 | Metroperitonitis | 2 |
| " crushed by fall | | Dropsy general | 6 | Meningitis | 9 |
| of house | 2 | " of pericardium | 1 | " spina bifida | 1 |
| " " by ma- | | Dyspepsia | 2 | " cerebro-spinal | 5 |
| chinery | 1 | Dysentery | 1 | " tubercular | 5 |
| Aneurism of arch of | | Embolism | 1 | Old age | 8 |
| aorta, | 1 | Enteritis | 8 | Oedema of glottis | 1 |
| Apthæ | 1 | Erysipelas | 3 | " pulmonum | 1 |
| Apoplexy | 5 | Exposure | 2 | Paralysis | 1 |
| Arachnitis | 1 | Fever congestive | 2 | Pericarditis | 1 |
| Asthma | 1 | " puerperal, | 4 | Peritonitis | 2 |
| Premature births | 12 | " remittent | 3 | Pleurisy | 1 |
| Still births | 54 | " scarlet | 7 | Pneumonia | 42 |
| Bowels, congestion of | 1 | " " & complicat's | 2 | " broncho | 1 |
| " ulceration of | 1 | " typhoid | 8 | " and complications | 3 |
| Brain, congestion of | 6 | Gangrene, dry | 1 | " typhoid | 3 |
| " inflammation of | 6 | Gastritis | 2 | Pyæmia | 1 |
| " softening of | 1 | Hæmorrhagica, puer- | | Rectum, ulceration of, | |
| " and lungs, con- | | peral | 1 | and diarrhœa | 1 |
| gestion of | 1 | Heart disease | 7 | Scrofula | 2 |
| Bronchitis | 6 | " dropsy of | 4 | " of hip-joint | 1 |
| " & heart dilitation | 1 | " fatty degeneration | 1 | Spina bifida | 1 |
| " capillary | 2 | " organic disease of | 1 | Spine disease, result of | |
| " chronic | 5 | " rheumatism of | 1 | injury | 1 |
| Cancer | 1 | " valvular disease | 3 | Stomach, gangrene of | 1 |
| " of breast | 1 | " mitral valves, in- | | Suicide by laudanum | 1 |
| " of pancreas | 1 | sufficiency of | 1 | " by drowning | 1 |
| " of womb | 1 | Hip-joint, disease of | 1 | Tabes mesenterica | 14 |
| " of stomach | 1 | Hemiplegia | 1 | Teething | 3 |
| Carries of dorsal spine | 1 | Hydrocephalus | 9 | " & complications | 1 |
| Consumption | 54 | " acute | 4 | Tetanus neonatorum | 1 |
| Convulsions | 61 | Inanition | 5 | Tumor of abdomen | 1 |
| " apoplectic | 1 | Jaundice | 1 | Uræmia | 1 |
| " uramic | 1 | Kidneys, uramic dis- | | Uterus, hemorrhage of | 2 |
| " puerperal | 2 | ease of | 1 | Whooping-cough | 4 |
| Croup | 15 | Kidneys, Bright's dis- | | " & complications | 1 |
| " diphtheritic | 1 | ease of | 4 | | |
| " membraneous | 4 | Liver, disease of | 1 | Total | 470 |

### AGES.

| | | | | | |
|---|---|---|---|---|---|
| Under 1 | 133 | 10 to 20 | 18 | 70 to 80 | 24 |
| 1 to 2 | 52 | 20 to 30 | 41 | 80 to 90 | 5 |
| 2 to 3 | 21 | 30 to 40 | 40 | 90 to 100 | |
| 3 to 4 | 21 | 40 to 50 | 43 | | |
| 4 to 5 | 13 | 50 to 60 | 27 | Total, | 470 |
| 5 to 10 | 13 | 60 to 70 | 15 | | |

| | | | | | |
|---|---|---|---|---|---|
| Males, | 259 | Females, | 211 | Total, | 470 |
| Single, | 313 | Married | 157 | Total, | 470 |
| White, | 458 | Colored, | 12 | Total, | 470 |

COMPARISON.

| | | |
|---|---|---|
| Deaths in Mar., 1871, 470 | Deaths in Mar., 1870, 538 | Decrease, 68 |
| Deaths in Feb., 1871, 401 | Increase, 69 | |

NATIVITY.

| | | | | | |
|---|---|---|---|---|---|
| Austria | 1 | England | 7 | New Brunswick | 1 |
| Bohemia | 3 | France | 1 | Norway | 7 |
| Canada | 5 | Germany | 66 | Russia | 1 |
| Chicago, Native | 76 | Holland | 2 | Switzerland | 1 |
| Chicago, Foreign | 167 | Ireland | 42 | Scotland, | 5 |
| U. S., other parts | 68 | Italy | 1 | Sweden | 9 |
| Denmark | 4 | New Foundland | 1 | Wales | 2 |
| | | | | Total, | 470 |

MORTALITY BY WARDS FOR THE MONTH.

| Wards. | Mortality. | Pop. in 1870. | One death in |
|---|---|---|---|
| 1 | 6 | 6,531 | 1088 |
| 2 | 13 | 14,338 | 1103 |
| 3 | 24 | 16,805 | 700 |
| 4 | 18 | 12,178 | 676 |
| 5 | 13 | 11,605 | 893 |
| 6 | 39 | 19,486 | 500 |
| 7 | 17 | 13,849 | 814 |
| 8 | 28 | 22,994 | 821 |
| 9 | 53 | 27,278 | 515 |
| 10 | 17 | 13,750 | 809 |
| 11 | 22 | 14,988 | 677 |
| 12 | 15 | 13,976 | 932 |
| 13 | 10 | 8,943 | 894 |
| 14 | 13 | 9,076 | 698 |
| 15 | 31 | 20,382 | 658 |
| 16 | 21 | 13,975 | 665 |
| 17 | 25 | 17,118 | 685 |
| 18 | 35 | 17,069 | 488 |
| 19 | 9 | 8,738 | 971 |
| 20 | 18 | 13,628 | 757 |

| Mortality. | |
|---|---|
| Accidents | 14 |
| County Hospital | 11 |
| Foundling Home | 6 |
| Home for Friendless | 2 |
| Hospital Alexian Brothers | 1 |
| Manslaughter | 1 |
| Marine Hospital | 1 |
| St. Joseph Orphan Asylum | 3 |
| St. Luke Hospital | 2 |
| Suicide | 2 |
| Total | 470 |

Mean Thermometer, 43°, Rain, 2.570 inches, Deaths daily, 15½.

AN apothecary in Boston has this notice put up conspicuously in his place of business: "We are pharmacists, but not physicians; we dispense medicines, but do not prescribe."—*Pacific Medical Journal.*

